# PCfun: a hybrid computational framework for systematic characterization of protein complex function

**DOI:** 10.1093/bib/bbac239

**Published:** 2022-06-21

**Authors:** Varun S Sharma, Andrea Fossati, Rodolfo Ciuffa, Marija Buljan, Evan G Williams, Zhen Chen, Wenguang Shao, Patrick G A Pedrioli, Anthony W Purcell, María Rodríguez Martínez, Jiangning Song, Matteo Manica, Ruedi Aebersold, Chen Li

**Affiliations:** Department of Biology, Institute of Molecular Systems Biology, ETH Zurich, Switzerland; CeMM Research Center for Molecular Medicine of the Austrian Academy of Sciences, Vienna, Austria; Quantitative Biosciences Institute (QBI) and Department of Cellular and Molecular Pharmacology, University of California, San Francisco, CA 94158, USA; J. David Gladstone Institutes, San Francisco, CA 94158, USA; Department of Biology, Institute of Molecular Systems Biology, ETH Zurich, Switzerland; Empa - Swiss Federal Laboratories for Materials Science and Technology, St. Gallen, Switzerland; Swiss Institute of Bioinformatics (SIB), Lausanne, Switzerland; Luxembourg Centre for Systems Biomedicine, University of Luxembourg, Esch-sur-Alzette Luxembourg; Collaborative Innovation Center of Henan Grain Crops, Henan Agricultural University, Zhengzhou 450046, China; Department of Biology, Institute of Molecular Systems Biology, ETH Zurich, Switzerland; Department of Biology, Institute of Molecular Systems Biology, ETH Zurich, Switzerland; Monash Biomedicine Discovery Institute and Department of Biochemistry and Molecular Biology, Monash University, Melbourne, VIC 3800, Australia; IBM Research Europe, Zurich, Switzerland; Monash Biomedicine Discovery Institute and Department of Biochemistry and Molecular Biology, Monash University, Melbourne, VIC 3800, Australia; Monash Data Futures Institute, Monash University, Melbourne, VIC 3800, Australia; IBM Research Europe, Zurich, Switzerland; Department of Biology, Institute of Molecular Systems Biology, ETH Zurich, Switzerland; Faculty of Science, University of Zurich, Switzerland; Department of Biology, Institute of Molecular Systems Biology, ETH Zurich, Switzerland; Monash Biomedicine Discovery Institute and Department of Biochemistry and Molecular Biology, Monash University, Melbourne, VIC 3800, Australia

**Keywords:** protein complex function, machine learning, gene ontology, natural language processing

## Abstract

In molecular biology, it is a general assumption that the ensemble of expressed molecules, their activities and interactions determine biological function, cellular states and phenotypes. Stable protein complexes—or macromolecular machines—are, in turn, the key functional entities mediating and modulating most biological processes. Although identifying protein complexes and their subunit composition can now be done inexpensively and at scale, determining their function remains challenging and labor intensive. This study describes Protein Complex Function predictor (PCfun), the first computational framework for the systematic annotation of protein complex functions using Gene Ontology (GO) terms. PCfun is built upon a word embedding using natural language processing techniques based on 1 million open access PubMed Central articles. Specifically, PCfun leverages two approaches for accurately identifying protein complex function, including: (i) an unsupervised approach that obtains the nearest neighbor (NN) GO term word vectors for a protein complex query vector and (ii) a supervised approach using Random Forest (RF) models trained specifically for recovering the GO terms of protein complex queries described in the CORUM protein complex database. PCfun consolidates both approaches by performing a hypergeometric statistical test to enrich the top NN GO terms within the child terms of the GO terms predicted by the RF models. The documentation and implementation of the PCfun package are available at https://github.com/sharmavaruns/PCfun. We anticipate that PCfun will serve as a useful tool and novel paradigm for the large-scale characterization of protein complex function.

## Introduction

Proteins are known to catalyze and control the majority of the reactions of cellular biochemistry [1]. Frequently, they exert their function only if they stably interact in precise stoichiometric ratios and defined steric arrangements with other proteins in the form of complex macromolecular structures, a notion that has been encapsulated in the term ‘modular cell biology’ [2]. With the advent of high throughput ‘omics’ technologies for the study of biological systems, it is now possible to accurately quantify and identify different types of biologically relevant molecules across various conditions at high throughput. However, associating the identified molecules with biological functions and phenotypes has remained challenging and requires a functional understanding of the molecules and their associations. Detailed biochemical and cell biological studies have identified the composition and even the atomic structures of numerous protein complexes with well-defined roles in a variety of fundamental biological processes [3], such as in their participation in transcriptional regulation [4–7], cell cycle control [8–10] signal transduction [11, 12] and protein homeostasis [13, 14]. Therefore, protein complexes can be considered essential agents and indicators of cellular functionality. Recent technical advances, particularly in mass spectrometry (MS) based proteomics, have greatly enhanced our capacity to determine the composition, stoichiometry and abundance of known protein complexes and identify new entities [15–19]. They also support the systematic identification of compositional or quantitative changes in complexes as a function of cellular state. These include methods such as biochemical fractionation MS [16–21], affinity purification MS [22, 23], cross-linking MS [24, 25] and limited proteolysis/thermal proteome profiling [26, 27]. Compared to the experimental detection of new protein complexes, the determination of their biochemical or cellular function has significantly lagged behind because functional characterization via experimentation for specific complexes is highly challenging. Given this challenge of characterizing the function of protein complexes, hypotheses regarding the functional roles in which a newly discovered protein complex participates are typically generated by a careful manual review of prior literature.

The standard approach to manual literature review for identifying the putative functions of a protein complex consists of first searching for publications and database entries describing the function of individual protein subunits and subsequent consolidation attempts to retrieve the information. An additional confounding consideration is the highly biased functional annotations toward well-studied individual proteins [28, 29]. Therefore, manual curation would present several limitations that make it inefficient and biased. Exhaustive literature curation for all proteins belonging to even a single complex can easily become prohibitively time-consuming due to the sheer volume of publications required to parse through. Given that the manual curation for retrieving high-confidence functional annotations of a single protein complex can be extremely laborious, performing such annotation on dozens or hundreds of novel entities discovered in large-scale complex centric proteomic fractionation experiments quickly becomes infeasible. Computational methods from text-mining and natural language processing—the fields concerned with computationally extracting information from unstructured natural language text—have been applied to a range of studies in the area of biomedical research and provide a promising avenue to address our task. These include protein-protein relations and functions [30–34], protein structure [35], protein localization [36] and gene-disease relationships [37]. However, to date, no computational tool designed to annotate the functions of protein complexes has been described.

To address this dearth of direct functional annotation methods for protein complexes, in this work, we integrate text-mining and machine-learning techniques into a hybrid computational framework, termed PCfun, which can be applied to large scale, complex-centric proteome experiments for predicting the function of protein complexes. At a high level, PCfun is developed based upon a word embedding generated from the machine reading of 1 million open access PubMed Central (PMC) articles, whereby both unsupervised and supervised machine learning algorithms were used to generate two separate lists of predicted functional Gene Ontology (GO; biological process, molecular function and cellular component) terms for a queried protein complex. Specifically, the unsupervised method was the nearest neighbor algorithm using cosine similarity between a protein complex query, and putative GO terms and the supervised machine-learning method was a model trained on the associations between protein complexes and their GO terms documented in the comprehensive resource of mammalian protein complexes (CORUM) database [38]. Hence, the unsupervised candidate list provides functional predictions solely based upon the word vector relationships observed within the embedding that are unbiased to protein complex-function associations, while the supervised candidate list tailors the annotations to relationships similar to the CORUM database. To leverage the insights provided by both approaches, we attempted to consolidate the two lists by leveraging the hierarchical structure of the GO by testing for the enrichment of certain supervised terms within the unsupervised list. An adapted leave-one-out cross-validation scheme was used to test the performance and suggested that PCfun achieves outstanding prediction performance with area under the receiver operator characteristic curve (AUC) values of 0.895, 0.927 and 0.957 for biological process, molecular function, and cellular component terms, respectively. In addition, we compared the prediction outcomes by PCfun and the GO annotations from the Complex Portal database [39] using protein complexes not documented in CORUM. Taken together, we anticipate that PCfun will serve as an accurate annotation tool for protein complex function and provide us with a better understanding of the functional roles of protein complexes in biological systems.

## Material and methods

### PCfun architecture overview

A word embedding was generated using the ‘fastText’ algorithm trained on approximately 1 million PubMed articles to build a vector representation space where biological semantic relationships were well reflected. To use this latent semantic space to predict the function of protein complexes, we extracted out sub-embeddings corresponding to all protein complexes and GO terms. To obtain the first predicted list of unsupervised GO annotations for a protein complex query, we stored the high-dimensional GO term sub-embeddings into *k-*d trees to easily retrieve predicted GO terms (nearest neighbors based on cosine similarity) when a protein complex was queried. To leverage a supervised learning paradigm for functional annotation, we generated datasets with binary-labeled relationships between protein complex embedding features and their GO annotations (also presented in their corresponding word embeddings) based on the CORUM database. We generated negative labels by sampling GO annotations that were not labeled for a particular protein complex. We then trained supervised machine learning classifiers on these generated datasets and tested the models on held-out portions of the generated datasets. For a new protein complex query (presented by its word embedding), we then tested it against every possible GO term with the supervised model and reported the terms that were predicted to positively associate with the protein complex query. This resulted in two predicted GO terms lists: one from the unsupervised approach and the other from the supervised model. To consolidate the information across the two models, we check for the enrichment of the unsupervised predicted GO terms within the child nodes of the predicted GO terms from the supervised classifier. For the functionally enriched terms (where information is agreed upon between the two approaches), we optionally plotted out the cut GO tree with the supervised algorithm’s predicted GO term and the multiple GO terms from the unsupervised method that were the children of the supervised model’s GO term. We then finally output the optional plots and the raw predicted lists from each method and the statistics indicating which terms were functionally enriched within the list by the Random Forest (RF) classifier.

### Text corpus generation and data processing

Approximately 1 million articles (including open-access full-text articles and their abstracts) were downloaded from PMC in February 2018. Note that these publications are not species/organism specific, which means the developed PCfun, built on the corpus, is a generic tool for protein complex function prediction. For processing the articles into a text corpus, we followed the text processing pipeline described in the study of Manica *et al.* [34]. All of the natural language queries were pre-processed by removing all punctuation characters, fixing Unicode mojibake and garbled HTML entities, and converting all uppercase characters into lowercase. To extract a single word vector for a natural language query (e.g. a protein complex or GO term name), individual component L2 normalized bi-gram vectors that built up the entire name were extracted from the embedding and then averaged. The final averaged vector of the component vectors of the name was once more L2 normalized and subsequently used as the final word vector for the natural language query.

### Databases for protein-complex annotations

For this work, we employed the CORUM database [38] as the main resource for the ground-truth annotation of protein complexes with GO terms, as CORUM is a compendium of manually curated and experimentally validated protein complexes for various organisms [38]. Annotations in CORUM for the function of protein complexes have been collected from various types of evidence, including experimental evidence (‘exp’), evidence from literature (‘lit’), known mammalian homologs (‘kmh’), high-throughput experiments (‘htp’), and predicted function (‘pred’). Here the ‘predicted function’ refers to the potential function suggested by the experimental results. In this work, we utilized annotations from all species in the CORUM database to keep as much information as possible for constructing an accurate supervised machine-learning model, given that the corpus we obtained from PubMed is not species/organism specific. In our study, the non-redundant 3414 core protein complexes (downloaded in March 2019) from the CORUM database were used.

In addition to CORUM, we utilized the well-annotated *Homo sapiens* protein complexes (downloaded in November 2019) from the Complex Portal database [39] to independently assess the prediction performance of PCfun. Similar to CORUM, the Complex Portal contains the protein complexes and their annotations of GO terms. As the Complex Portal has fewer protein complexes documented, we did not use it for the model training purpose. For the independent test, only the protein complexes annotated as ‘physical interaction evidence used in manual assertion’ with the evidence code ‘ECO:0000353’ coupled with experimental evidence from the IntAct database [40] were retained. To objectively benchmark the performance of PCfun on the Complex Portal, we further removed those protein complexes from the Complex Portal that had a subunit overlap of larger than 50% compared to the complexes in the CORUM database. As a result, the numbers of protein complexes from the Complex Portal for the independent test were in total 34, of which 34, 31 and 33 protein complexes have biological processes, molecular functions and cellular component annotations, respectively.

### Semantic similarity calculation between GO terms

To compare the GO terms predicted by PCfun and the annotations from Complex Portal, we did not perform the direct comparison/matching of the GO terms due to the low number of intersecting GO terms between CORUM and Complex Portal. Instead, we applied the semantic similarity introduced by Wang *et al* [41] to perform the comparison. This method considers biological meanings and hierarchical relationships in the GO direct acyclic graph (DAG) structure of the given GO term pair. Any GO term pairs with the semantic similarity ≥0.5 were considered similar. When comparing the predicted GO terms by PCfun and the annotations from Complex Portal for each protein complex, we first calculated the similarities for all possible GO term pairs between the PCfun outputs and Complex Portal annotations. The pairs with a similarity score ≥ 0.5 were selected, and the unique Complex Portal GO terms (*N*) in the pairs were counted. Then the percent coverage of the predicted GO terms to the Complex Portal annotation for the particular protein complex was calculated using $Coverage=(N/M)\times 100$, where *M* denotes the number of GO terms annotated in Complex Portal for the particular protein complex.

### GO term enrichment analysis of predicted functional terms

This step aims to systematically and comprehensively combine the two predicted GO term lists in each category (i.e. biological process, molecular function, and cellular component) by RF and the *k*-d tree for a given protein complex, respectively, because this shortlist of terms predicted by RF that recovers CORUM database well but tends to predict broader GO terms for a protein complex. Given the predicted GO term list by RF with the size *N* (*N* ≤ 10), we supplemented the information from this list with the list of GO terms (i.e. the nearest neighbors) obtained directly by querying the GO term sub-embedding. To accomplish this, we developed a functional enrichment analysis pipeline based on the hypergeometric test to assess if all the child nodes of the GO term by RF are significantly enriched in the predicted GO terms by the *k*-d tree, using the following formula:

$$ p={f}_{\mathrm{hypergeometric}}\left(x-1,M,n,N\right), $$

where *M* denotes the number of total GO terms for a particular GO term class, *n* denotes the number of child terms of a parent GO term plus the parent term predicted by the supervised classifier that exists within the specific GO term class, where *N* denotes the sample size which is the mean number of GO terms required for the nearest neighbor list to recover all GO terms annotated for a protein complexes (biological process = 11 044, molecular function = 5213, and cellular component = 1896, respectively), and *x* denotes the number of child terms for a particular supervised term that exists in the set of the sample size list. The function ${f}_{\mathrm{hypergeometric}}$ was the survival function for the hypergeometric distribution as implemented in the SciPy package. If the child nodes/terms are significantly enriched in the predicted list by the *k*-d tree, 10 top-ranked terms based on cosine similarity from the *k*-d tree list are selected. We could therefore obtain a ‘combined’ predicted list that not only accurately recovers the CORUM database but also supplements the list by RF using the detailed GO terms predicted by the *k*-d tree. To visualize the results, PCfun plots a GO tree structure of the predicted GO term by RF (in green) and the 10 top-ranked GO terms by the *k*-d tree (in purple) to demonstrate the hierarchical relationships of these terms. For the cellular component category and the combination of the two lists from the *k*-d tree and the RF model by functional enrichment analysis, we also considered adding the overlap of cellular component annotations of all the subunits to the final outputs. The cellular component annotations for each subunit were downloaded from the QuickGO database [42].

### PCfun prediction output organization

In total, there are six output lists (two for each GO category) for a given protein complex generated by PCfun. For each GO category (i.e. biological process, molecular function and cellular component), one list contained the RF predictions and the top 10 significantly enriched GO terms by the *k*-d tree, while the other lists provided the top-20 GO terms by the *k*-d tree only. In addition, for each RF predicted term, a GO DAG structure is plotted to illustrate the hierarchical relationships between the RF prediction and the top 10 significantly enriched terms from the *k*-d tree.

## Results

### Architecture of PCfun for predicting the function of protein complexes

**Figure 1:**
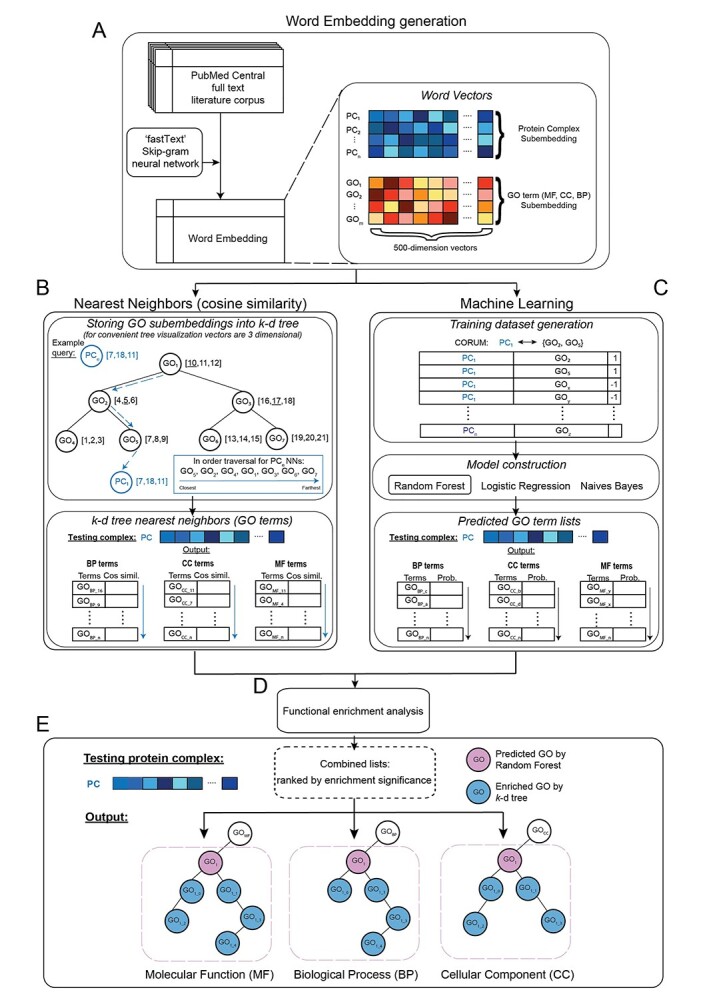
The overall framework of the PCfun methodology. (**A**) A word embedding containing a 500-dimensional vector for each word was first generated based on the open-access full-text articles and their abstracts using the ‘fastText’ with a skip-gram model. Based on the word embedding, two machine-learning algorithms were used, specifically (**B**) a *k*-d tree for nearest-neighbor search and (**C**) a supervised RF model for PC association with molecular function (MF), biological process (BP), and cellular component (CC), respectively. A simplified *k*-d tree example is shown in the top panel of (**B**). To combine the outputs of the two models, function enrichment analysis (**D**) was performed. PCfun utilizes the enrichment analysis and GO DAG structure (**E**) to represent and visualize the predicted GO terms for a given protein complex. The testing protein complex (i.e. ‘PC’) is used to illustrate the procedures of PCfun.

The development of PCfun involved three main steps, which are schematically illustrated in Figure 1. Collectively these steps constitute a workflow that uses automated text mining and structured, curated information on biological functions to predict the function of complexes of interest. The following describes the steps of PCfun in detail.

#### Word embedding

**Figure 2:**
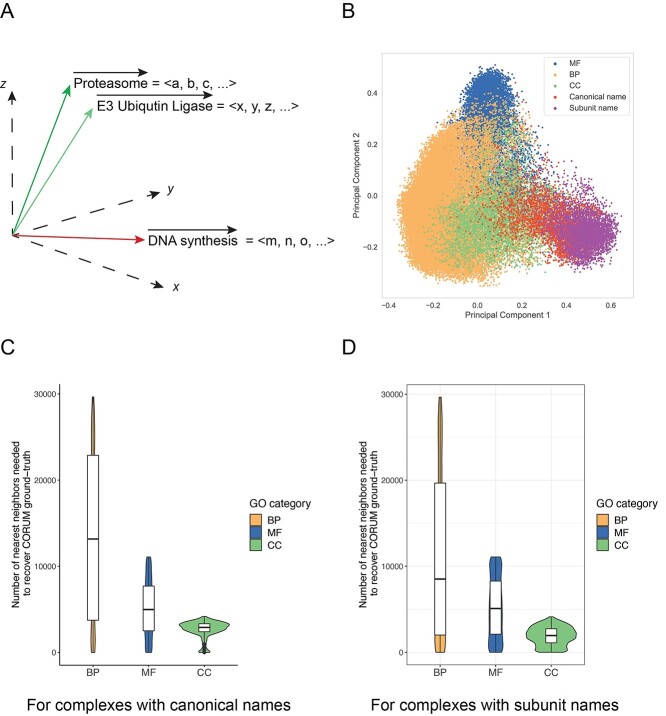
Shortlisting the GO terms for protein complexes using the word embedding and *k*-d tree. (**A**) A graphical illustration of the vectors of phrases ‘Proteasome,’ ‘E3 Ubiquitin Ligase’ and ‘DNA synthesis’ in a 3D space using simplified vector representation, for example, $\lt a,b,c,\dots \gt$ denotes the numerical word vector for the natural language query ‘Proteasome’. (**B**) The principal component analysis results of different types of sub-embeddings, including molecular function, biological process, cellular component, protein complexes with canonical names, and subunit names. (**C**) The numbers of nearest neighbors required from the *k*-d tree search outputs for protein complexes using canonical names to cover the CORUM ground truth. (**D**) The numbers of nearest neighbors required from the *k*-d tree search outputs for protein complexes using subunit names to cover the CORUM ground truth.

The first step is the generation of the word embedding (Figure 1A). Approximately 1 million open access articles were downloaded from the PMC Repository, and the texts were processed (‘Supplementary Methods’) as described in Manica *et al* [34] to populate a text corpus. A word embedding of 129 459 words was constructed using the text corpus and the ‘fastText’ [43] package with a skip-gram model. In this matrix, each word is represented using a 500-dimension continuous real-valued vector (‘Supplementary Methods’). Using this property of the constructed word embedding, we next extracted sub-embeddings consisting of all protein complex and GO terms (split into biological process, molecular function, and cellular component classes). Since the ‘fastText’ skip-gram model was trained using character bi-grams, the vector for a protein complex or a GO term was obtained by averaging the 500-dimensional vector embeddings of the individual character bi-grams make up the natural language protein complex name or the GO term. For example, for the GO term ‘positive regulation of viral transcription,’ fastText would first split the term into bi-grams (e.g. ‘po,’ ‘os,’ ‘si,’ ‘it,’ ‘ti,’ ‘iv,’ ‘ve’), obtain each bi-gram’s 500-dimensional vector embedding, and then average all of the component bi-gram vectors to obtain a final 500-dimensional vector embedding for the whole query GO term. The same process is performed for calculating a vector for a protein complex using either the complex canonical name or the names of the complex subunit components (‘Supplementary Methods’). Figure 2A presents a graphic illustration of the approach we used to generate the word vectors.

#### Model creation for functional prediction

The second step aims to construct the models for functional annotation of protein complex queries. We employed two strategies capable of returning ranked protein complex—GO associations: (i) an unsupervised nearest-neighbor approach illustrated in Figure 1B, and (ii) a supervised machine learning approach displayed in Figure 1C. The first algorithm is agnostic to the question of functional annotation of protein complexes and was based solely upon contextual relationships provided by the vector representation derived from text mining. The second approach is a tailored approach trained specifically to recover functional terms for a protein complex query. The rationale for using two distinct approaches is that they are likely to produce complementary and potentially if combined, more informative outputs. As illustrated in Figure 1B, we built a *k*-d tree (*k*-dimensional tree) [44], a space-partitioning structure for storing the sub-embeddings’ vectors of GO terms to support the rapid application of the nearest neighbor algorithm. These algorithm shortlisted GO terms ranked by cosine similarity between the queried protein complex vector and each word vector for a GO term to recover CORUM’s ground-truth annotations of each protein complex. The supervised machine-learning models (Figure 1C), on the other hand, learns from the experimentally verified and highly curated protein complex—GO term associations and are therefore able to accurately cover the ground truth in the CORUM database. We constructed and evaluated four widely applied machine-learning algorithms, including RF [45], Logistic Regression (LR) [46], and Naïve Bayes (NB; with Gaussian and Bernoulli distributions) [47] classifiers. A ranked list of GO term annotations was generated by both the unsupervised *k*-d tree algorithm (Figure 1B) and the supervised machine learning models (Figure 1C).

#### Functional enrichment analysis

The third step aims at combining the prediction outcomes from the RF and the *k*-d tree via a functional enrichment analysis approach (Figure 1D). An optional visualization of a GO DAG structure for functionally enriched predicted GO terms is performed to represent the contextual information of predicted GO terms of biological process, molecular function and cellular component, respectively (Figure 1E). Given a protein complex of interest, PCfun first applies the two models to generate two prediction lists using the *k*-d tree and RF, assesses the agreement between the two prediction outcome lists via the functional enrichment analysis, and then visualizes the GO DAG structure (‘Material and Methods’ and ‘Supplementary Methods’).

### Benchmarking the performance of PCfun

In this section, we systematically evaluated the prediction performance of PCfun. We first separately assessed the predictive ability of the two modules of PCfun, specifically the unsupervised *k*-d tree and the RF model, for annotating protein complex function. We further independently compared the prediction outputs from the enrichment analysis of PCfun with the functional annotations documented in the Complex Portal database [39].

#### The word embedding and k-d tree facilitate the ranking of potential GO terms for protein complexes

A useful property of word embedding is that words with related semantic meanings have corresponding word vectors that exist closer to each other in the word vector space—as measured by the cosine similarity (i.e. same orientation)—than words with a different meaning. Therefore, one can find similar words to an input query word by simply finding the nearest neighbors of the input query word vector. To aid in rapid nearest-neighbor calculations for these large sub-embeddings, we stored each sub-embedding into a *k*-d tree, allowing us to efficiently retrieve similar word vectors to the input query vector. We performed principal component analysis of the word vectors for each extracted sub-embedding of different types, including biological process vectors, molecular function vectors and cellular component vectors, and the protein complex vectors with the two naming schemes, including ‘*canonical name*’ and ‘*subunit name*’ (‘Supplementary Methods’). Figure 2B demonstrates that the sub-embeddings’ word vectors of each type are well clustered, indicating the reliable quality of the word embedding.

To access the prediction power of the *k-d* tree, we measured the ability of each GO term class sub-embedding to recover the ground-truth functional annotations for a protein complex from CORUM. This was accomplished by recording the number of nearest neighbors (i.e. GO terms for a protein complex query vector ranked by their cosine similarity) required to recover 100% of the ground-truth functional annotations for the input protein complex query. We hypothesized that the results might change depending on the name used to represent a protein complex. Additionally, considering that *de novo* detected protein complexes will not be characterized with an accepted name, we proposed a subunit naming scheme for a protein complex that would still allow for the functional annotation of even newly identified protein complexes by PCfun. We tested the two protein complex naming schemes’ (i.e. ‘*canonical name*’ and ‘*subunit name*’) sub-embeddings. Figure 2C, D indicate that the sub-embeddings required, on average 13,487, 5119, 2692 and 11,044, 5214, 1894 nearest neighbors to recover the ground truth for biological process, molecular function, and cellular component categories using the canonical names and subunits names, respectively. It is evident that to recover CORUM’s ground-truth annotations, a large number of nearest neighbors are required. Despite the low performance on our recovery benchmark metric, we suspected that the top nearest neighbors may actually still contain useful information that went beyond the annotation within the CORUM database. We therefore subsequently performed a manual literature search based on the top nearest neighbor GO terms for certain protein complexes and observed that the predicted GO terms were actually still quite informative and were recovering known biological knowledge.

To illustrate this effect, we chose the protein complex termed ‘Mothers against decapentaplegic homolog 2 (SMAD2)-Mothers against decapentaplegic homolog 4 (SMAD4)-Forkhead box protein H1 (FAST1)-Homeobox protein TGIF1 (TGIF)-Histone deacetylase 1 (HDAC1) complex, Transforming growth factor (TGF) (beta) induced’ for a manual literature review comparison to the *k*-d tree nearest-neighbor results. We used the subunit naming scheme [‘smad4 tgif1 smad2 hdac1 foxh1 (fast1)’] for the generation of its corresponding word vector and then queried the vector into the biological function, molecular function and cellular component sub-embedding *k*-d trees. While the *k*-d trees required 27,400, 2182 and 990 nearest neighbor terms in the biological function, molecular function and cellular component trees, respectively, to recover the six CORUM annotated GO terms (DNA topological change; negative regulation of transcription, DNA-templated; DNA binding; transforming growth factor beta receptor signaling pathway; chromosome organization; nucleus), for this protein complex, the top returned *k*-d tree nearest neighbors (Supplementary Table S1) still provided relevant GO terms that had related biological meanings. For example, the top 10 nearest neighbors for the biological function category are related to the Transforming Growth Factor Beta (TGFβ) or bone morphogenic protein response. According to Massague *et al* [48], the SMAD proteins accumulate in the nucleus to execute transcriptional control in response to TGFβ signal transduction and may be co-activated or co-repressed by various DNA-binding co-factors. We observed that ‘negative regulation of smad protein signal transduction’ was the 8th nearest neighbor term for the queried protein complex vector, which recovers the role of the co-repressor activity of HDAC1 and TGIF that act to repress the transcriptional control of the activated SMAD2:SMAD4 subcomplex localized in the nucleus [49, 50]. Thus, a manual literature review indicated co-repression activity in response to TGFβ signal transduction, which the *k*-d tree recovered within its top 10 neighbors. For the complex ranking of GO terms by the *k*-d tree for this protein complex, please refer to Supplementary Files S2 (available under the ‘Supplementary Files_Tables’ on the PCfun GitHub repository (https://github.com/sharmavaruns/PCfun) for more details. Despite the poor ability of the *k*-d tree to recover CORUM’s ground-truth annotations, the top nearest neighbor results still provided significant insight into the relevant biology.

#### Supervised machine-learning models substantially improved the performance of ground-truth recovery of GO terms in CORUM

To improve the performance of ground-truth recovery of CORUM, we implemented supervised machine-learning classifiers based on the word vectors for a ‘protein complex-GO association’ pair (termed ‘PC-GO’). In our study, the annotated association of a PC-GO term was regarded as a positive sample, whereas the synthetic pairs of a protein complex and other GO terms that were not associated in CORUM were regarded as negative ones. As the negative samples significantly outnumbered the positive samples in the resulting datasets, we generated five different training datasets with randomly selected negative samples and all positives for each protein complex to ensure an equal distribution of positive and negative samples for training the classifier. This process was conducted for both naming schemes. With the training datasets, we assessed the performance of three machine-learning classification algorithms, specifically RF, LR, and NB with a Gaussian or Bernoulli prior (NB_Gauss or NB_Bernoulli, respectively), through the adapted ‘protein complex’-leave-one-out cross-validation strategy using standard performance measures (‘Supplementary Methods’).

**Figure 3:**
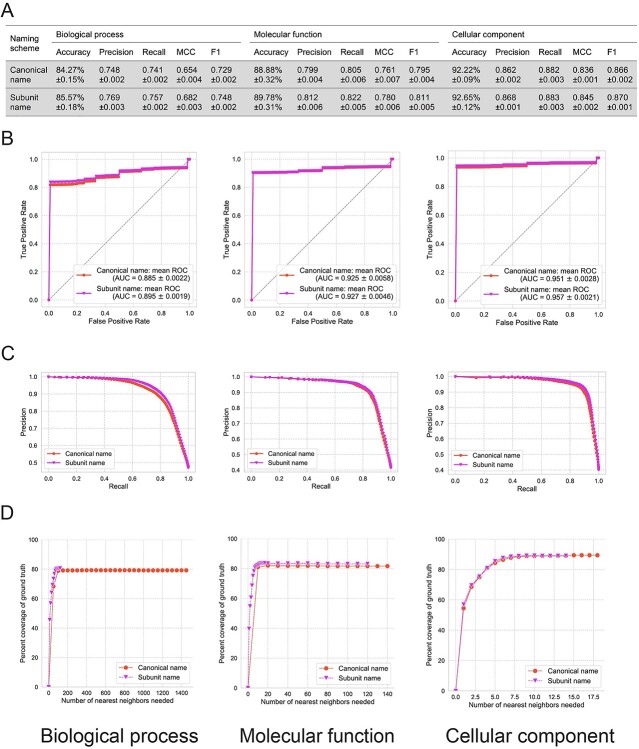
Prediction performance of RF model trained on the ground-truth protein complex – GO term annotations in the CORUM database for biological process, molecular function, and cellular component using canonical and subunit naming schemes for protein complexes, respectively, including (**A**) performance measures of RF models; (**B**) ROC curves and mean AUC values; (**C**) the precision-recall curves of the RF models via the adapted protein complex leave-one-out cross-validation, and (**D**) the numbers of predicted GO terms by the RF models to recover the CORUM database annotations.

Across these classifiers tested, RF consistently performed best as measured by all performance metrics (Figure 3, Supplementary Table S2, and Supplementary Figures S1 and S2) and achieved a robust performance across the two naming schemes. For example, via the ‘protein complex’-leave-one-out cross-validation strategy, the RF classifier achieved AUC values of 0.885 and 0.895 for biological function, 0.925 and 0.927 for molecular function, and 0.951 and 0.957 for the cellular component category for protein complexes with ‘*canonical names*’ and ‘*subunit names,*’ respectively. In addition, we also observed that the resulting GO term lists predicted by the RF classifiers were able to significantly reduce the number of nearest neighbors needed to recover the majority of the ground-truth GO term annotations for a protein complex when compared to the nearest-neighbor results from querying the *k*-d tree (Figure 3D). For example, for protein complexes with subunit names, the RF classifier predicted terms were able to recover 80.5, 83.6 and 89.2% of CORUM’s ground-truth in 102, 49 and 11 positively predicted terms for biological process, molecular function, and cellular component, respectively.

**Figure 4:**
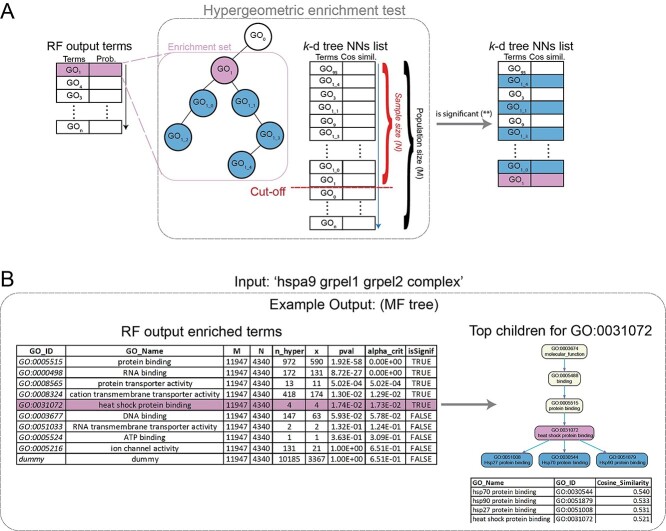
Functional enrichment analysis of the combined prediction lists from the *k*-d tree and the supervised RF model. (**A**) Hypergeometric enrichment test based on the RF prediction list. For each term in the list, all the child nodes of the term were collected and used for the statistical significance test with the terms from the *k*-d tree. If significant, the top-10 terms from the *k*-d tree, the child nodes of the RF term, were selected and visualized in the GO DAG structure. (**B**) An example of prediction results for the protein complex ‘Stress-70 protein, mitochondrial (hspa9) GrpE protein homolog 1, mitochondrial (grpel1) GrpE protein homolog 2, mitochondrial (grpel2) complex’ illustrates the enrichment analysis procedure.

While the RF classifiers performed well to recover the ground truth as documented in the CORUM database, the supervised approach’s performance may belie the inherent bias to the database that it was trained on. Although protein complexes within CORUM have been extensively studied and the GO term annotations have been manually curated, there is an obvious right skew in the frequency of GO terms with low to middle depth, based on the GO DAG structure. From Supplementary Figure S3, we observed that the logged frequency of a particular GO term (i.e. the number of times a GO term has been annotated in CORUM) versus each GO term’s depth in the GO DAG structure reveals a biased annotation distribution for GO terms in CORUM. For example, the biological process term ‘Regulation of transcription DNA templated,’ molecular function term ‘DNA binding’ and cellular component term ‘Nucleus’ were annotated in 233, 278 and 702 protein complexes in the CORUM database. Such over-annotated GO terms would lead to biased machine-learning algorithms favorably toward them. Therefore, to address the biases of the dataset that the RF classifier was trained on, we supplemented the predicted terms from the RF classifier with the predicted nearest neighbors from the *k*-d tree. A graphical illustration of the combination of the RF and *k*-d tree prediction lists together and an example is shown in Figure 4. It can be concluded that RF achieved the best performance in recovering the CORUM annotations for a given protein complex. In summary, the prediction results of the *k*-d tree and RF model are complementary and thus highlight the necessity of systematically and statistically combining the predictive outcomes from the *k*-d tree and RF models.

#### Independent test demonstrates divergent GO term predictions by PCfun compared to complex portal

**Figure 5:**
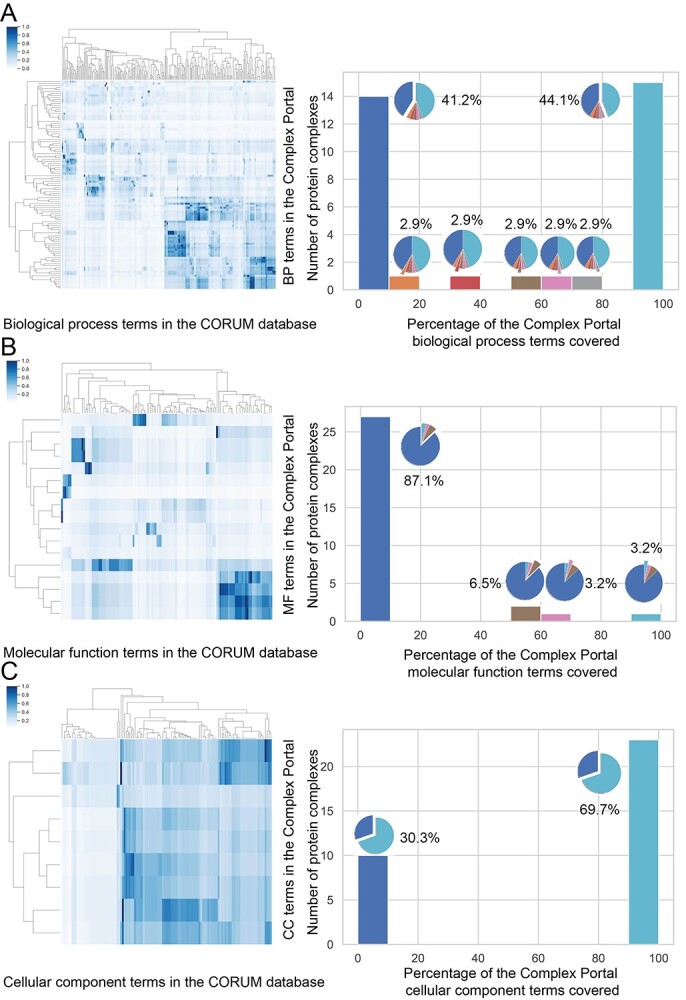
Semantic similarity comparison of (**A**) biological process, (**B**) molecular function and (**C**) cellular component terms for the Complex Portal annotations with the CORUM annotations on identical protein complexes (left panel) and PCfun predictions on non-overlapping protein complexes (right panel). The right panel illustrates the percentage breakdowns of the predicted similar biological process and molecular function terms by PCfun to the Complex Portal annotations, respectively. Any GO term pairs between PCfun predictions and Complex Portal annotations with the semantic similarity ≥ 0.5 were considered similar.

To assess the consistency of PCfun predictions and the experimental annotations of a protein complex, we compared our predicted terms with the annotations provided in the Complex Portal. We first identified that only 110 annotated human protein complexes were shared by the CORUM and Complex Portal databases with identical subunit composition. We subsequently interrogated the semantic similarity of their biological process, molecular function and cellular component terms between the two databases (‘Material and Methods’). The heatmaps of the pairwise similarity scores using the method reported by Wang *et al* [41] are shown in the left panel of Figure 5. The average semantic similarity scores of biological process, molecular function and cellular component categories were 0.40, 0.34 and 0.54, respectively, suggesting that even for the same protein complex with precisely the same subunit composition the annotations of CORUM and Complex Portal are dissimilar. We examined the numbers of identical GO terms used for each overlapping protein complex more stringently. As a result, on average less than one GO term across all categories (0.22 biological process term, 0.05 molecular function term and 0.13 cellular component term, respectively) was shared per protein complex between CORUM and Complex Portal. This means that the annotations for protein complexes are extremely divergent across the two databases, making it challenging for PCfun (built on CORUM) to accurately cover the GO annotations in the Complex Portal. We then sought to gauge the approximate similarity of GO terms predicted by PCfun with Complex Portal annotations. The right panels of Figure 5 show the comparison between predicted biological process, molecular function and cellular component terms by PCfun and the Complex Portal annotations for the non-overlapping protein complexes (i.e. with <50% of overlapping subunits). For a biological process, as shown in Figure 5A, 15 (approximately 44.1%) non-overlapping complexes covered 90–100% of similar terms compared to Complex Portal biological process annotations, while 14 complexes (41.2%) had divergent predictions (i.e. coverage between 0 and 10%) compared to the annotations in the Complex Portal. Similarly, for cellular components (Figure 5C), approximately 69.7% (23) of the complexes covered 90–100% of similar terms compared to Complex Portal cellular component annotations of 30.3% (10). In contrast, PCfun demonstrated highly divergent predictions for the molecular function category, with only 1 (3.2%) complex covering 90–100% of similar terms and 27 (87.1%) complexes demonstrating 0–10% coverage of similar terms when compared to Complex Portal’s annotations. The low coverage of PCfun prediction for the molecular function category might be related to the poor similarity between the molecular function annotations between the CORUM and Complex Portal databases (the left panel of Figure 5B). It is noteworthy that different studies and databases may have divergent annotations for a protein or protein complex. Despite the generally low semantic similarities of all the biological processes, molecular functions and cellular component terms between the two databases, PCfun still demonstrates its ability to recover the ground truth of the databases and to provide novel biological knowledge for a given protein complex.

## Discussion

Although computational methods for gene function prediction [51–53] have significantly broadened our understanding of single proteins, the extrapolation from the functions of the constituent proteins to the function of a protein complex is non-trivial, and the scarcity of high confidence functional annotation of protein complexes is impeding molecular biology research in several ways. First, functional annotation of protein complexes is inconsistent between databases. Although CORUM and Complex Portal offer experimentally and manually validated functional annotations for protein complexes (i.e. the ground truth), the literature-based functional annotations for protein complexes shared across the databases can be highly dissimilar depending on the database chosen. Second, we argue that because complexes with already annotated functions are preferentially cited in the literature, an increasing fraction of research is focused on already well-known entities, introducing a strong bias in contents. Third, some proteins are multifunctional and perform unique tasks in the context of different protein complexes, thereby highlighting that the function of protein complexes is not simply the aggregate of their subunits’ functions [54–56]. As a case in point, a brief examination of the GO terms annotated for whole protein complexes in the CORUM database compared to each individual subunit’s GO term annotations in the QuickGO database [42] showed that 2155 (61.4%), 319 (9.1%) and 169 (4.8%) protein complexes contained at least one novel biological process, molecular function, or cellular component term, respectively, that was not annotated for any individual subunit’s QuickGO entry. In other words, certain proteins may participate in emergent functionality when assembled in a macromolecular complex that would be non-obvious based upon the known functions of the individual protein complex’s subunits. Therefore, we argue that it is of great importance to employ computational techniques to assist with the prediction of protein complex function and provide useful insight and guidance for experiments to characterize the functions of protein complexes.

As discussed in the ‘Results’ section, one issue during the construction of the machine-learning models is the biased functional annotations in the CORUM database, as shown in Supplementary Figure S3. Therefore, the predictive power of the RF model in PCfun is limited to the CORUM annotations, demonstrating that it is crucial to combine the ‘non-biased’ prediction results of the unsupervised *k*-d tree method with RF predictions via enrichment analysis for PCfun to deliver non-biased predicted functions for a given protein complex. Another noteworthy issue is the negative data for training the supervised RF model of PCfun. This problem has been brought to attention and discussed in our previous studies [57, 58]. In this study, similar issues occurred when constructing the negative PC–GO associations in the training datasets (‘Supplementary Methods’). Theoretically, there would be many negative protein complex-GO pairs, some of which might be mislabeled. Compared to the traditional supervised machine-learning models, positive-unlabeled learning [57, 59] only requires positive and unlabeled (i.e. either positive or negative) training samples to build reliable predictors with competitive prediction performance and, therefore, can be considered as an alternative option for tackling this issue.

Further limitations to the PCfun word embedding strategy for annotating protein complex function lie in the featurization of the protein complex itself, which currently does not leverage any structured database information on the protein interactome network for achieving an informative embedding. Currently, the word embedding only leverages inferred relationships between character *n*-grams to build word-embedding relationships between complicated multi-character strings (e.g. protein complex names). However, protein complexes can also be viewed as functional subgraphs of a protein-protein interaction network [60, 61], and the network connectivity itself may explicitly encode important functional information about the protein complex that is not leveraged by the current word embedding methodology.

To address these limitations regarding (1) the bias of training/assessing an algorithm on just the CORUM and Complex Portal databases and (2) the current exclusion of structured protein network information in the protein complex embeddings, we propose the following future work. Firstly, a systematic analysis of the shared and diverging information between the current protein complex–functional annotation databases would be extremely informative for formally detecting biases in the databases and defining confidence metrics for certain annotations. Thus, a consolidated gold-standard database of all such protein complex–functional annotations could be constructed, thereby enabling much more accurate and generalizable models to be built for this task. Regarding the featurization limitation, we believe that tailoring embedding representations from constructed knowledge graphs such as from Himmelstein *et al* [62], where both gene and GO annotations have been incorporated into the database, along with additional functional information such as pathways and diseases, could act as a sophisticated source for alternate embeddings that could be well suited for this protein complex-functional annotation task. Furthermore, state-of-the-art modeling approaches such as Graph Neural Networks could be used, which are capable of learning highly expressive embedding representations of graph-structured data and have been applied to protein function prediction [63], and human microbe–drug associations embeddings [64]. These interactomes or structure-based protein complex embeddings could then be tailored for their functional annotations using concepts from representation learning. Alternatively, it would be useful to compare the performance of word or interactome-based network embeddings to traditional matrix factorization latent space learning techniques where our task would be link prediction in a bipartite graph that connects protein complexes to GO terms [65, 66].

Overall, we believe PCfun is the first-in-class word embedding-based functional annotator for protein complexes and can be applied to broad biological and personalized medicine applications. It could be possible to compare the functional differences of subunit composition by interrogating the prediction outputs of PCfun for a protein complex across different biological/medical conditions, given that the differential analysis of protein composition is possible through the development of new techniques and computational methods [17, 20, 21, 67]. Taken together, we anticipate that PCfun can be exploited as an instrumental computational approach for the identification of novel functions and the demarcation of functional alterations of the complex-centric proteomic across different biological conditions.

**Key Points**

- PCfun is the first computational framework focusing on rapid and accurate annotations of functional Gene Ontology (GO) terms for protein complexes by integrating word embedding and supervised machine learning approaches.
- The word embedding was constructed using the fastText algorithm on approximately 1 million PubMed articles, and an unsupervised *k*-d tree using the word embedding was trained to rank the GO terms.
- We separately trained a Random Forest using the manually curated CORUM protein complex database for supervised learning of protein complex–GO relationships.
- We anticipate that PCfun will serve as an instrumental computational tool for annotating the functional GO terms of protein complexes.

## Supplementary Material

Sharma_et_al_supplementary_revised_bbac239

## Author Biographies

**Varun S. Sharma** is a PhD student at the Center of Molecular Medicine in Vienna, Austria. His research is focused on developing AI-based causal representation methods for modeling perturbation biology and network medicine.

**Andrea Fossati** is a postdoctoral fellow in the Quantitative Bioscience Institute at the University of California, San Francisco, and J. David Gladstone Institutes, San Francisco.

**Rodolfo Ciuffa** is a postdoctoral alumnus from the laboratory of Ruedi Aebersold at the ETH Zurich. His research interests include proteomics-based modeling of protein–protein interactions and cellular signaling.

**Marija Buljan** is a group leader at the Swiss Federal Laboratories for Materials Science and Technology and her group is also affiliated with the Swiss Institute of Bioinformatics (SIB).

**Evan G. Williams** is an associate professor at the Luxembourg Centre for Systems Biomedicine at the University of Luxembourg. His research interests include the cellular management of gene transcription and translation, population genetics, and inferring causality from gene co-expression networks.

**Zhen Chen** is a professor at the Collaborative Innovation Center of Henan Grain Crops, Henan Agricultural University, China. His research interests include protein bioinformatics, machine learning and analysis of next-generation sequencing data.

**Wenguang Shao** is an associate professor at the School of Life Sciences and Biotechnology, Shanghai Jiao Tong University. His research mainly focuses on computational proteomics, immunopeptidomics, systems biology and big data analysis.

**Patrick G.A. Pedrioli** is the group leader of the data science group of the ETH Zurich Personalized Health and Related Technologies (PHRT) Clinical Proteotype Analysis Center. His research interests are centered around the roles played by proteoforms in health and disease.

**Anthony W. Purcell** is a professor and group leader in the Monash Biomedicine Discovery Institute and Department of Biochemistry and Molecular Biology, Monash University, Australia. He is currently an NHMRC Principal Research Fellow. He specializes in targeted and global quantitative proteomics of complex biological samples, with a specific focus on identifying targets of the immune response and host–pathogen interactions.

**Maria Rodriguez Martinez** is the technical leader of Computational Systems Biology at IBM Research Europe (Switzerland) and an associated member of the Department of Biology at ETH Zurich. Her research interests include computational immunology and artificial intelligence for personalized medicine.

**Jiangning Song** is an associate professor and group leader in the Biomedicine Discovery Institute and Department of Biochemistry and Molecular Biology, Monash University, Melbourne, Australia. He is also affiliated with the Monash Centre of Data Science, Faculty of Information Technology, Monash University. His research interests include bioinformatics, computational biology, machine learning, data mining and pattern recognition.

**Matteo Manica** is a research staff member in Accelerated Discovery at IBM Research Europe–Zürich. He is currently working on AI-driven chemical synthesis automation and the development of multimodal generative models for material science. He obtained his doctoral degree jointly at IBM Research and the Institute of Molecular Systems Biology, ETH Zürich.

**Ruedi Aebersold** is a professor Emeritus in molecular systems biology at ETH Zurich. His research has focused on exploring the proteome and the determination of functional consequences of alterations of the proteome.

**Chen Li** is a research fellow in the Biomedicine Discovery Institute and Department of Biochemistry of Molecular Biology, Monash University. His research interests include systems proteomics, immunopeptidomics, personalized medicine and experimental bioinformatics.
