# Supplementary material for: PCfun: a hybrid computational framework for systematic characterization of protein complex function
Source: Brief Bioinform. 2022 Jun 21;23(4):bbac239. doi: 10.1093/bib/bbac239 (PMC9310514; doi:10.1093/bib/bbac239)
Supplement: Sharma_et_al_supplementary_revised_bbac239 [file sharma_et_al_supplementary_revised_bbac239.docx]

**PCfun: a hybrid computational framework for systematic characterization of protein complex function**

Varun S. Sharma^1,2^, Andrea Fossati^3,4^, Rodolfo Ciuffa^1^, Marija Buljan^5,6^, Evan G. Williams^7^, Zhen Chen^8^, Wenguang Shao^1^, Patrick G.A. Pedrioli^1^, Anthony W. Purcell^9^, María Rodríguez Martínez^10^, Jiangning Song^9,11,*^, Matteo Manica^10,*^, Ruedi Aebersold^1,12,*^, and Chen Li^1,9,*^

^1^Department of Biology, Institute of Molecular Systems Biology, ETH Zürich, Switzerland

^2^CeMM Research Center for Molecular Medicine of the Austrian Academy of Sciences, Vienna, Austria

^3^Quantitative Biosciences Institute (QBI) and Department of Cellular and Molecular Pharmacology, University of California, San Francisco, CA 94158, USA

^4^J. David Gladstone Institutes, San Francisco, CA 94158, USA

^5^Empa - Swiss Federal Laboratories for Materials Science and Technology, St. Gallen, Switzerland

^6^Swiss Institute of Bioinformatics (SIB), Lausanne, Switzerland

^7^Luxembourg Centre for Systems Biomedicine, University of Luxembourg, Esch‐sur‐Alzette Luxembourg

^8^Collaborative Innovation Center of Henan Grain Crops, Henan Agricultural University, Zhengzhou 450046, China

^9^Monash Biomedicine Discovery Institute and Department of Biochemistry and Molecular Biology, Monash University, Melbourne, VIC 3800, Australia

^10^IBM Research Europe, Zürich, Switzerland

^11^Monash Data Futures Institute, Monash University, Melbourne, VIC 3800, Australia

^12^Faculty of Science, University of Zürich, Switzerland

*Correspondence: [Jiangning.Song@monash.edu](mailto:Jiangning.Song@monash.edu) (J. Song), [TTE@zurich.ibm.com](mailto:TTE@zurich.ibm.com) (M. Manica), [aebersold@imsb.biol.ethz.ch](mailto:aebersold@imsb.biol.ethz.ch) (R. Aebersold), and [Chen.Li@monash.edu](mailto:Chen.Li@monash.edu) (C. Li).

**Supplementary Methods**

**Word embedding and similarity calculations**

Word embedding, a method developed in the fields of text mining and natural language processing, refers to a class of approaches that embed natural language texts into high-dimensional, continuous real-valued vector representations [1]. The word embedding in this study was achieved by training the unsupervised version of ‘fastText’ [2] with a skip-gram model on the corpus. Briefly, the skip-gram model attempts to minimize the negative sum of the log probability that a word will exist within the context of a target word. The fastText unsupervised training parameters used were defaults except for 500-dimensions for the embedding layer, a context window of size 9, and the usage of bi-grams. These parameters have been chosen since they led to superior performance in the study of Manica *et al* [1]*.* After training, the word vectors were normalized to a unitary norm. We utilized the cosine similarity between two vectors, which measures the cosine of the angle between the two vectors as the normalized dot product of the two vectors.

**Calculating sub-embeddings of topics and nearest neighbors**

We extracted sub-embeddings for protein complex names (extracted from CORUM) and GO terms (extracted from GO resource split into biological process, molecular function and cellular component classes). For example, to obtain the GO terms most similar to a protein complex query in embedding space, calculation of the nearest neighbors to the protein complex query vector within the extracted GO term sub-embedding space would provide the most similar GO term word vectors to the protein complex query. A nearest-neighbor calculation involves calculating distances between vectors within the sub-embedding of interest to the query vector and then sorting the neighbors by their similarity by descending order. The calculation for nearest neighbors can be quite time intensive depending on the size of the embedding due to the time requirements for pairwise calculation of the distances between each vector in the sub-embedding and the query vector. Therefore, we stored the sub-embedding vectors into a pre-computed *k*-d tree (*k*-dimensional tree), which is a space-partitioning data structure that stores the vectors into buckets determined by hyperplane splits over each dimension of the vector [3]. Therefore, calculation of *n*-nearest neighbors to a query vector requires only placement of the query vector into its corresponding location within the pre-calculated *k*-d tree and then subsequent querying of its ancestors of the tree until the closest *n*-nearest neighbors have been calculated.

**Gene name extraction for protein complex subunits**

As each unique natural language query has its own unique word vector, it is important to standardize the natural language name used for each protein complex when extracting its word vector. A protein complex can be either represented as its documented name, as written into the protein complex database, or as its subunits’ gene names strung together. In this study, we tested the performance of the algorithms using two different naming schemes: (1) *canonical name* (as documented in CORUM), and (2) *subunit name* (composed by UniProt gene names of each subunit). To obtain the gene names of the subunits, we extracted the subunits of the protein complexes from CORUM and queried the UniProt database (downloaded in May 2019) [4] for their corresponding gene names. We then represented the protein complex name with its text pre-processed subunits’ gene names, strung together with spaces demarcating each individual gene name. Due to the fact that there might exist multiple names for a single gene, only its canonical name was extracted and used in our model. Gene names were extracted by downloading the UniProt FASTA-formatted sequence file, with respect to the appropriate species, which was then subsequently parsed for each relevant UniProt ID - gene name pair. The resulting work has tested the performance of the algorithm when using each naming scheme independently from each other. Importantly, use of the subunit UniProt gene name scheme also allows for greater flexibility as one can still gain functional insight into a newly detected protein complex even if the complex has not been officially named yet.

**Preparation of protein complex - GO (PC-GO) pair datasets for supervised learning**

To enable the accurate prediction of protein complex function annotations, we have formulated our task as a supervised binary classification problem, for which we created a labelled dataset based on the CORUM annotations. To create the labelled dataset, we first extracted PC-GO term pairs and then labelled each pair as positive if its annotation was observed in the CORUM database. The GO terms and their DAG structures were collected from the Gene Ontology Resources platform [5, 6]. To label the negatives, we first generated a pool of all possible negative PC-GO pairs to sample from by taking the GO terms (split by biological process, molecular function and cellular component categories) that were used in CORUM and not annotated for a particular protein complex. Since only a few GO terms were annotated for each protein complex, this negative sample pool significantly outnumbered the positive sample pool. This issue of having a huge number of negatives compared to positive labels is a common problem shared by a variety of bioinformatics studies and have been discussed in the recent studies of Li *et al* [7, 8]. To build an unbiased supervised classifier, it is common practice to train on an approximately equal distribution of positive and negative labels. To ensure this, we randomly selected an approximately equal number of negative PC-GO pairs from the pool of negatives as there were positives for each protein complex. This random selection was repeated five times, resulting in five training datasets, where only negative samples were different.

**Supervised machine-learning classifiers**

Supervised binary classification was performed with the RF [9], LR [10], and NB (with Gaussian and Bernoulli distributions, respectively) [11] classifiers with the default parameters. The feature space consisted of 1,000-dimensional vectors (500-dimensional protein complex vector prepended to a 500-dimensional GO term vector) with each vector corresponding to a PC-GO pair labelled with either positive or negative. *RF* classification uses the ensemble of decision trees that randomly bootstraps over the training data and features for each decision tree in order to classify the input vector space. After construction of the random decision trees, the classifier outputs a class membership (positive or negative for binary classification) prediction dependent on the community-wide vote from the random trees constructed. LR utilizes the logistic function for the binary classification of a dependent variable. This method outputs a probability score ranging from 0 to 1 where values of >0.5 are considered to be of the positive class membership. *NB classifiers* are probabilistic classifiers built upon Bayesian statistics. These classifiers attempt to learn the distributional fit for each labelled class and accordingly assign probability values for each class when given a new input vector. The priors we tested were the Gaussian and Bernoulli distributions. Briefly, the Gaussian distribution assumes that the continuous data values are distributed according to a normal distribution, whereas the Bernoulli distribution assumes that the features are independent binary variables and can be considered as a special case of the binomial distribution. These two classifiers are termed NB_Gauss and NB_Bernoulli in the following sections, respectively. For PCfun’s machine learning classifier predicted terms we used a majority voting scheme over the five datasets to provide an averaged predicted probability for each GO term. For this majority vote, we equally weighted the contribution of each dataset’s model to achieve a final combined probability score for particular GO terms. If the combined probability score was >0.5, the GO term was classified as a positive term, otherwise classified as a negative.

**Performance assessment of supervised binary classification**

To assess the predictive performance of the supervised binary classifiers, we introduced an adaptation on traditional leave-one-out cross-validation, which we termed ‘protein complex’-leave-one-out cross validation. The ‘protein complex’-leave-one-out cross validation first pre-removed every row that had the particular protein complex being tested (including both positive and negatively labelled rows). Afterward, the model was then trained on the remaining dataset and tested on the pre-removed protein complex of interest’s rows. This complex-wise evaluation strategy was applied to each protein complex in the dataset. To measure the performance, we used widely established performance metrics used in a variety of bioinformatics and computational biology studies [12-14], including accuracy, AUC (Area Under the Curve), precision, recall, MCC (Matthews Correlation Coefficient) [15], and F1 score. All metrics reported in this study are the average of five datasets of ‘protein complex’-leave-one-out cross-validation. For plotting the Receiver Operating Characteristic (ROC) curves, we followed the recommendations by the scikit-learn package using interpolation (refer to the scikit-learn tutorial). These scores are calculated based upon the elementary scores of true positives (*TP*), true negatives (*TN*), false positives (*FP*), and false negatives (*FN*). The formulas for each performance metric are provided as follows:

$$Accuracy=\frac{TP + TN}{TP+ FP+FN+TN},$$

$$Preicision=\frac{TP}{TP+FP}\boldsymbol{,}$$

$$Recall\boldsymbol{=}\frac{TP}{TP+FN}\boldsymbol{,}$$

$$MCC\boldsymbol{=}\frac{TP\times TN-FP\times FN}{\sqrt{\left( TP+FN \right)\left( TP+FP \right)\left( TN+FP \right)(TN+FN)}}\boldsymbol{,}$$

$$F1=2\times\frac{precision\times recall}{precision+recall}\boldsymbol{.}$$

**DATA AND CODE AVAILABILITY**

**Data and code availability statement**

***Data availability***

The full-text articles and their abstracts (in the non-commercial use collection) were extracted from the PubMed Central under a Creative Commons or similar license. The training dataset and the validation test (i.e., the PC-GO association) were obtained from the CORUM database [16] and the Complex Portal database [17], respectively. The full GO lists were downloaded from the Gene Ontology Resource platform [5, 6]. For evaluation purposes, we downloaded the GO annotations for individual proteins from the QuickGO database [18].

***Code availability***

PCfun is an open-access software and is freely available for academic use under the ‘Academic Free License v3.0’. The source code, user instruction, and example inputs can be downloaded from <https://github.com/sharmavaruns/PCfun>.

**Supplementary Tables**

**Table S1.** Top 10 GO terms shortlisted by the *k*-d tree for the protein complex “SMAD2-SMAD4-FAST1-TGIF-HDAC1 complex, TGF(beta) induced”.

| Number | GO term | Cosine distance | Cosine similarity | GO ID |
| --- | --- | --- | --- | --- |
| Biological process | | | | |
| 1 | jun phosphorylation | 0.90142032 | 0.52592264 | GO:0007258 |
| 2 | common partner smad protein phosphorylation | 0.91515871 | 0.52214994 | GO:0007182 |
| 3 | smad protein signal transduction | 0.9206383 | 0.52066024 | GO:0060395 |
| 4 | pathway restricted smad protein phosphorylation | 0.92724758 | 0.51887469 | GO:0060389 |
| 5 | regulation of histone h3 t3 phosphorylation | 0.93482421 | 0.51684282 | GO:2000281 |
| 6 | regulation of smad protein signal transduction | 0.93509793 | 0.51676971 | GO:0007184 |
| 7 | histone h3 t3 phosphorylation | 0.93731143 | 0.51617927 | GO:0072355 |
| 8 | negative regulation of smad protein signal transduction | 0.94274921 | 0.51473448 | GO:0060392 |
| 9 | regulation of pathway restricted smad protein phosphorylation | 0.9430544 | 0.51465363 | GO:0060393 |
| 10 | negative regulation of histone h3 k27 trimethylation | 0.94409184 | 0.51437899 | GO:1902465 |
| Molecular function | | | | |
| 1 | smad binding | 0.81312028 | 0.55153539 | GO:0046332 |
| 2 | i smad binding | 0.88015558 | 0.53187088 | GO:0070411 |
| 3 | r smad binding | 0.89042146 | 0.52898257 | GO:0070412 |
| 4 | co smad binding | 0.89897215 | 0.52660067 | GO:0070410 |
| 5 | bmp (bone morphogenic protein) binding | 0.93230116 | 0.51751767 | GO:0036122 |
| 6 | activin binding | 0.95586228 | 0.51128344 | GO:0048185 |
| 7 | histone deacetylase activity h3 k14 specific | 0.96414003 | 0.50912867 | GO:0031078 |
| 8 | bmp receptor binding | 0.96599249 | 0.50864894 | GO:0070700 |
| 9 | transcription corepressor binding | 0.97184839 | 0.50713838 | GO:0001222 |
| 10 | bmp receptor activity | 0.97334295 | 0.50675429 | GO:0098821 |
| Cellular component | | | | |
| 1 | pdx1 pbx1b mrg1 complex | 0.84789228 | 0.54115709 | GO:0034978 |
| 2 | gata2 tal1 tcf3 lmo2 complex | 0.86410241 | 0.53645121 | GO:0070354 |
| 3 | rgs6 dnmt1 dmap1 complex | 0.86604966 | 0.53589142 | GO:0070313 |
| 4 | gata1 tal1 tcf3 lmo2 complex | 0.87113766 | 0.53443422 | GO:0070353 |
| 5 | heteromeric smad protein complex | 0.8795976 | 0.53202877 | GO:0071145 |
| 6 | smad protein complex | 0.88390432 | 0.53081252 | GO:0071141 |
| 7 | maml3 rbp jkappa icn1 complex | 0.89328291 | 0.52818308 | GO:0071179 |
| 8 | maml1 rbp jkappa icn1 complex | 0.89389121 | 0.52801343 | GO:0002193 |
| 9 | homomeric smad protein complex | 0.90011405 | 0.5262842 | GO:0071143 |
| 10 | fhl2 creb complex | 0.90109935 | 0.52601144 | GO:0034980 |

**Table S2.** Performance comparison of the Logistic Regression (LR) and Naïve Bayes (NB_Gauss and NB_Bernoulli) classifiers trained on the CORUM database via the adapted protein-complex leave-one-out cross-validation, for biological process, molecular function and cellular component categories, respectively.

| Naming scheme | Biological process | | | | | |  | Molecular function | | | | | |  | Cellular component | | | | | |
| --- | --- | --- | --- | --- | --- | --- | --- | --- | --- | --- | --- | --- | --- | --- | --- | --- | --- | --- | --- | --- |
|  | Acc.^1^ | AUC | Precision | Recall | MCC | F1 |  | Acc. | AUC | Precision | Recall | MCC | F1 |  | Acc. | AUC | Precision | Recall | MCC | F1 |
| **LR** | | | | | | | | | | | | | | | | | | | | |
| Canonical name | 73.05%  ±0.47% | 0.764  ±0.005 | 0.613  ±0.008 | 0.638  ±0.010 | 0.419  ±0.009 | 0.602  ±0.009 |  | 81.2%  ±0.33% | 0.842  ±0.005 | 0.69  ±0.007 | 0.708  ±0.006 | 0.599  ±0.007 | 0.688  ±0.007 |  | 87.55%  ±0.34% | 0.914  ±0.005 | 0.785  ±0.005 | 0.801  ±0.007 | 0.735  ±0.007 | 0.785  ±0.006 |
| Subunit name | 73.4%  ±0.30% | 0.77  ±0.002 | 0.623  ±0.006 | 0.642  ±0.008 | 0.428  ±0.004 | 0.609  ±0.007 |  | 82.36%  ±0.55% | 0.858  ±0.004 | 0.71  ±0.005 | 0.727  ±0.005 | 0.625  ±0.007 | 0.708  ±0.004 |  | 87.71%  ±0.40% | 0.919  ±0.004 | 0.789  ±0.007 | 0.808  ±0.007 | 0.741  ±0.009 | 0.79  ±0.008 |
| **NB_Gauss** | | | | | | | | | | | | | | | | | | | | |
| Canonical name | 64.66%  ±0.36% | 0.702  ±0.004 | 0.503  ±0.009 | 0.547  ±0.012 | 0.249  ±0.008 | 0.497  ±0.009 |  | 73.89%  ±0.375% | 0.783  ±0.006 | 0.594  ±0.014 | 0.648  ±0.013 | 0.467  ±0.010 | 0.602  ±0.012 |  | 83.15%  ±0.10% | 0.867  ±0.006 | 0.687  ±0.003 | 0.704  ±0.005 | 0.638  ±0.002 | 0.687  ±0.004 |
| Subunit name | 64.82%  ±0.39% | 0.699  ±0.001 | 0.485  ±0.007 | 0.529  ±0.009 | 0.245  ±0.008 | 0.479  ±0.008 |  | 73.54%  ±0.672% | 0.796  ±0.007 | 0.581  ±0.012 | 0.641  ±0.009 | 0.46  ±0.014 | 0.593  ±0.010 |  | 82.47%  ±0.98% | 0.872  ±0.005 | 0.661  ±0.022 | 0.674  ±0.022 | 0.619  ±0.022 | 0.66  ±0.022 |
| **NB_Bernoulli** | | | | | | | | | | | | | | | | | | | | |
| Canonical name | 64.89%  ±0.36% | 0.701  ±0.004 | 0.500  ±0.009 | 0.534  ±0.008 | 0.252  ±0.009 | 0.489  ±0.007 |  | 72.56%  ±0.658% | 0.784  ±0.005 | 0.563  ±0.013 | 0.622  ±0.011 | 0.438  ±0.013 | 0.575  ±0.011 |  | 81.98%  ±0.21% | 0.871  ±0.006 | 0.645  ±0.002 | 0.650±  0.0 | 0.604  ±0.002 | 0.642  ±0.001 |
| Subunit name | 64.33%  ±0.64% | 0.694  ±0.003 | 0.475  ±0.007 | 0.515  ±0.008 | 0.236  ±0.012 | 0.468  ±0.008 |  | 72.46%  ±0.640% | 0.796  ±0.006 | 0.558  ±0.004 | 0.621  ±0.0 | 0.437  ±0.014 | 0.572  ±0.003 |  | 82.18%  ±0.21% | 0.875  ±0.002 | 0.647  ±0.001 | 0.654  ±0.0 | 0.609  ±0.002 | 0.644  ±0.001 |

^1^Acc: Accuracy


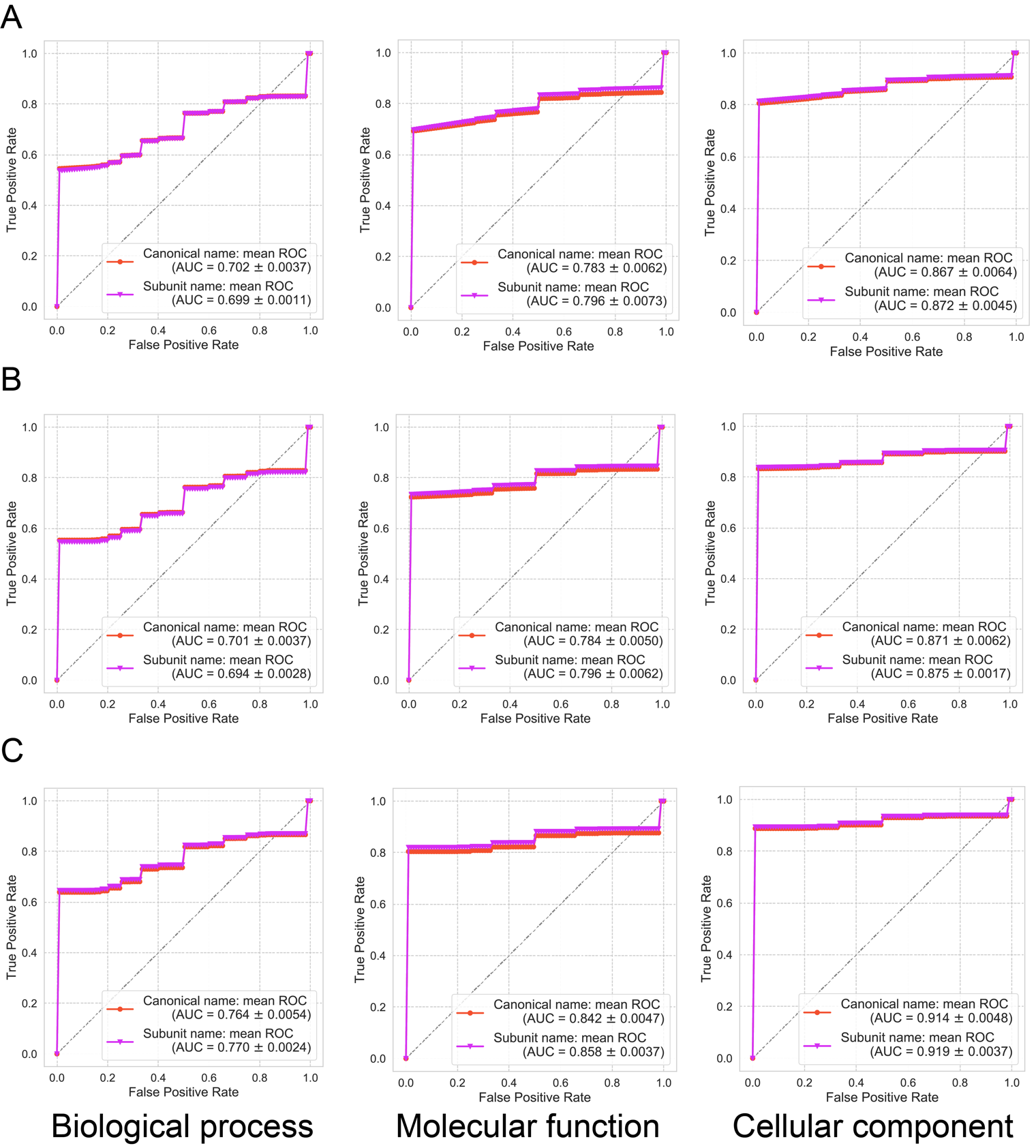


**Figure S1.** ROC curves and the average AUC values of (A) NB_Gauss, (B) NB_Bernoulli and (C) LR models on biological process, molecular function and cellular component categories via the adapted protein complex leave-one-out cross-validation. These models were trained using the CORUM ground-truth PC-GO associations, where the protein complexes were represented using either canonical (red line) and subunit (pink line) naming schemes.


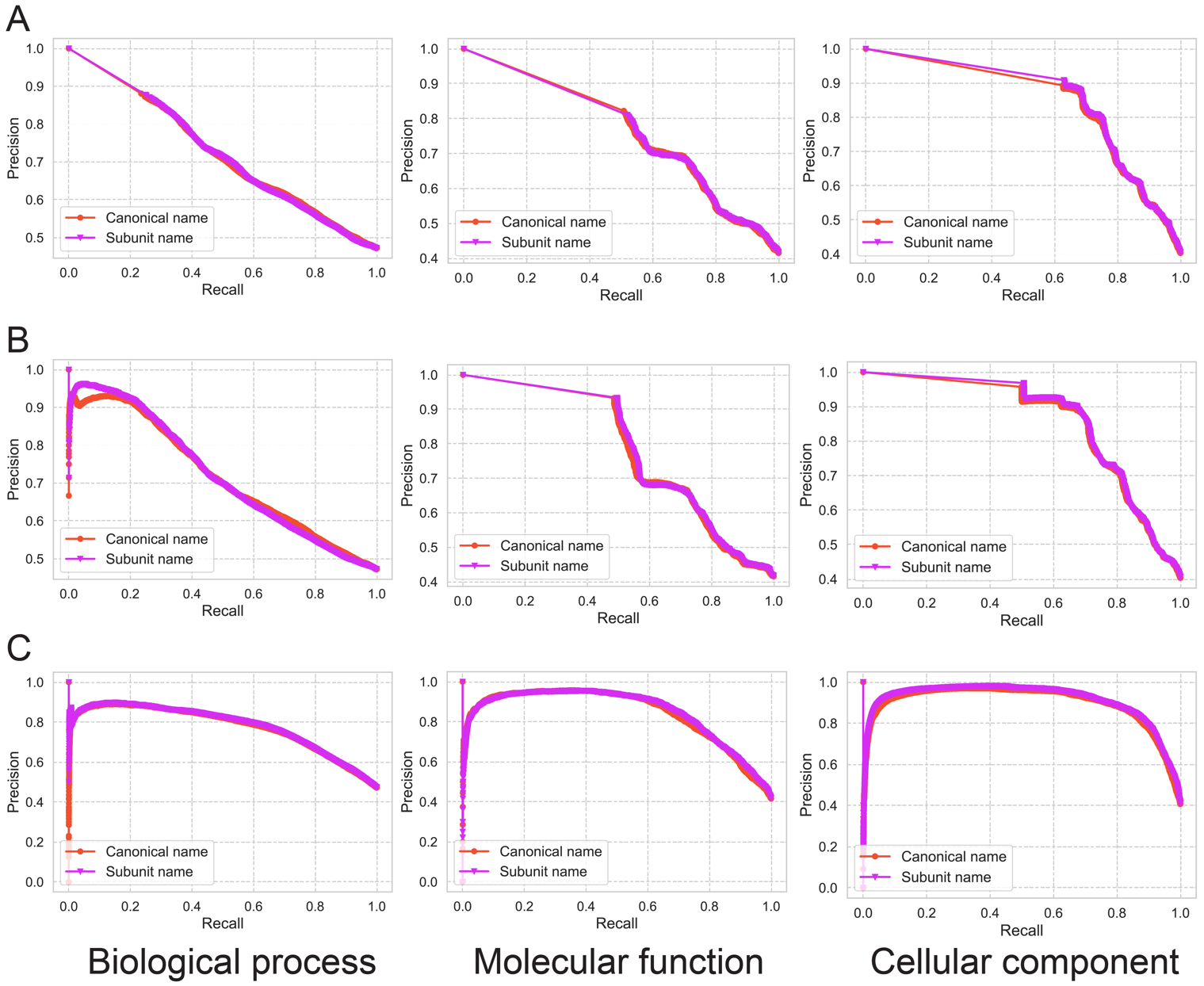


**Figure S2**. Precision-recall curves of (A) NB_Gauss, (B) NB_Bernoulli and (C) LR models on biological process, molecular function and cellular component categories via the adapted protein complex leave-one-out cross-validation, where the protein complexes were represented using both canonical (red line) and subunit (pink line) naming schemes.


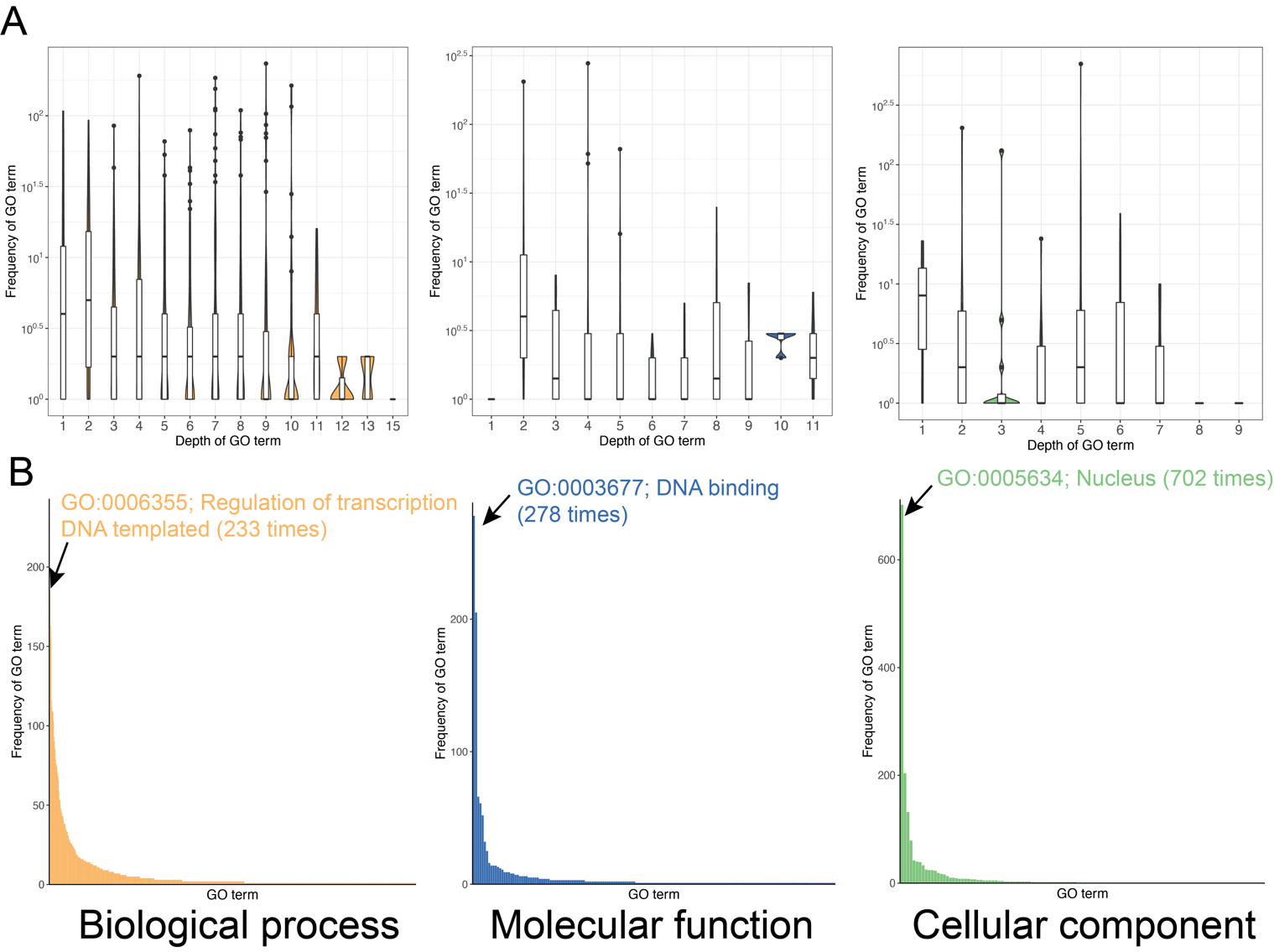


**Figure S3.** Statistical analyses of the annotated GO terms in the CORUM database, including (A) distributions of the depth of biological process, molecular function and cellular component terms in the GO DAG structures and (B) frequencies of biological process, molecular function and cellular component terms that were assigned to the CORUM protein complexes. The top over-annotated biological process, molecular function and cellular component terms are respectively indicated.

References

1. Manica M, Mathis R, Cadow J, Martínez MR: **Context-specific interaction networks from vector representation of words.** *Nature Machine Intelligence* 2019, **1:**10.

2. Joulin A, Grave E, Bojanowski P, Mikolov T: **Bag of Tricks for Efficient Text Classification.** In *The 15th Conference of the European Chapter of the Association for Computational Linguistics; Valencia, Spain*. Association for Computational Linguistics; 2017: 5.

3. Freidman JH, Bentley JL, Finkel RA: **An Algorithm for Finding Best Matches in Logarithmic Expected Time.** *ACM Transactions on Mathematical Software* 1977, **3:**209.

4. UniProt C: **UniProt: a worldwide hub of protein knowledge.** *Nucleic Acids Res* 2019, **47:**D506-D515.

5. Ashburner M, Ball CA, Blake JA, Botstein D, Butler H, Cherry JM, Davis AP, Dolinski K, Dwight SS, Eppig JT, et al: **Gene ontology: tool for the unification of biology. The Gene Ontology Consortium.** *Nat Genet* 2000, **25:**25-29.

6. The Gene Ontology C: **The Gene Ontology Resource: 20 years and still GOing strong.** *Nucleic Acids Res* 2019, **47:**D330-D338.

7. Li F, Wang Y, Li C, Marquez-Lago TT, Leier A, Rawlings ND, Haffari G, Revote J, Akutsu T, Chou KC, et al: **Twenty years of bioinformatics research for protease-specific substrate and cleavage site prediction: a comprehensive revisit and benchmarking of existing methods.** *Brief Bioinform* 2018.

8. Li F, Zhang Y, Purcell AW, Webb GI, Chou KC, Lithgow T, Li C, Song J: **Positive-unlabelled learning of glycosylation sites in the human proteome.** *BMC Bioinformatics* 2019, **20:**112.

9. Breiman L: **Random forests.** *Machine Learning* 2001, **45:**5-32.

10. Lecessie S, Vanhouwelingen JC: **Ridge Estimators in Logistic-Regression.** *Applied Statistics-Journal of the Royal Statistical Society Series C* 1992, **41:**191-201.

11. Zhang H: **The Optimality of Naïve Bayes.** In *THE SEVENTEENTH INTERNATIONAL FLORIDA ARTIFICIAL INTELLIGENCE RESEARCH SOCIETY CONFERENCE*. AAAI; 2004

12. Fossati A, Li C, Uliana F, Wendt F, Frommelt F, Sykacek P, Heusel M, Hallal M, Bludau I, Capraz T, et al: **PCprophet: a framework for protein complex prediction and differential analysis using proteomic data.** *Nat Methods* 2021, **18:**520-527.

13. Li F, Li C, Marquez-Lago TT, Leier A, Akutsu T, Purcell AW, Ian Smith A, Lithgow T, Daly RJ, Song J, Chou KC: **Quokka: a comprehensive tool for rapid and accurate prediction of kinase family-specific phosphorylation sites in the human proteome.** *Bioinformatics* 2018, **34:**4223-4231.

14. Manavalan B, Basith S, Shin TH, Wei L, Lee G: **mAHTPred: a sequence-based meta-predictor for improving the prediction of anti-hypertensive peptides using effective feature representation.** *Bioinformatics* 2019, **35:**2757-2765.

15. Matthews BW: **Comparison of the predicted and observed secondary structure of T4 phage lysozyme.** *Biochim Biophys Acta* 1975, **405:**442-451.

16. Giurgiu M, Reinhard J, Brauner B, Dunger-Kaltenbach I, Fobo G, Frishman G, Montrone C, Ruepp A: **CORUM: the comprehensive resource of mammalian protein complexes-2019.** *Nucleic Acids Res* 2019, **47:**D559-D563.

17. Meldal BHM, Bye AJH, Gajdos L, Hammerova Z, Horackova A, Melicher F, Perfetto L, Pokorny D, Lopez MR, Turkova A, et al: **Complex Portal 2018: extended content and enhanced visualization tools for macromolecular complexes.** *Nucleic Acids Res* 2019, **47:**D550-D558.

18. Binns D, Dimmer E, Huntley R, Barrell D, O'Donovan C, Apweiler R: **QuickGO: a web-based tool for Gene Ontology searching.** *Bioinformatics* 2009, **25:**3045-3046.
